# Preventive Measures in Relation to War Neuroses in the United States Army

**Published:** 1918-08

**Authors:** John H. W. Rhein


**Preventive Measures in Relation to War Neuroses in the U.S. Army Cantonments in America.** Major John H. W. Rhein, M. R. C. The speaker said that by prophylactic measures in relation to War Neuroses he refers more especially to those which were and are being employed in the training camps in the United States with a view of preventing wastage in the Army with special references to ineffectives due to War Neuroses. Of these, the acute neuroses following causes, both commotional and emotional and other etiological factors form a large part. While these manifestations occur among men not predisposed to nervous break-downs before their entrance into the army, experience with the Allied Armies as well as our own army shows, and this statement will be generally accepted, that many of the cases exhibiting neuroses after exposure to front line experiences are psychoneurotics to begin with.

The recognition of this type of individual, that is, the psycho-neurotic or the one predisposed to the development of neuroses, upon his admission to the cantonments and during his training period, and the prompt discharge from the Army, were believed to be important measures tending to increase the safety, efficiency, and economy of military service in the A. E. F.

The object then of these preventive measures was to eliminate from the Army at an early date those who were nervously unstable, those who were actually suffering from neuroses, and those who appeared to be predisposed to nervous breakdowns. The function of these preventive measures included, however, also the elimination of those individuals which showed other evidences of nervous or mental disorders, organic or functional, within certain limits.

The examinations were made by members of boards, or by groups of neuropsychiatrists, who conducted surveys or who examined cases referred to them.

Upon admission to camps the medical examiners distributed among the camp infirmaries examined all the drafted Army, and a number of the men were recognized at the first examination as belonging to the group of cases which should be referred to the special neuro-psychiatric examiners. These cases were then first sent to the Special Board of Medical Examiners, who in turn referred them to that member of the board who was a neuro-psychiatrist, for examination and recommendations. Later this same board disposed of numbers of this type of cases which were referred for discharge on S. C. D. Surveys were also systematically made and these were intended to include the entire personnel : officers, enlisted men, members of classes, officers training corps, and applicants for enlistment, with a view to selecting cases for special examinations. To this end, consultations were made with regimental surgeons and company commanders as well as the making of personal observations by neuropsychiatrists of soldiers when they were collected together in groups for inspections of various sorts such as vaccinations, inoculations, heart, lung, orthopedics, or when grouped together for the special purpose of inspections by neurologists and psychiatrists. In addition to this, a neuro-psychiatrist visited the various organizations, observed the behavior of the men during drills, and at the same time consulted with medical officers, instructors, company commanders, and sergeants and selected in this way subjects for examination by boards or by the special neuropsychiatric examiner.

Medical officers, dental surgeons, instructors, hospital sergeants, barrack sergeants and Company Commanders were instructed to refer cases for examination by the neuro-psychiatrist who showed certain characteristics such as irritability, seclusiveness, sulkiness, depression, stupidity, personal uncleanliness, resentfulness under discipline, inability to be disciplined, sleep walking, nocturnal eneuresis, or those who became the boobs, cranks, goats, and queer sticks of the company. Characteristics such as queerness, peculiarities, and idiosyncrasies which are often the early symptoms of mental diseases, were searched for.

Causes for rejection included organic nervous disease, unless the symptoms were no longer in operation and not likely to recur, or unless residual symptoms were present which did not interfere with a satisfactory performance of military duties; for example, paralysis of unimportant muscles after poliomyelitis or slight hypertonicity after infantile hemiplegia were not sufficient to reject. Hemiplegia after infancy always was a cause for rejection, as was also Existing neuritis.

Emphasis was laid on the importance of recognizing the early symptoms of tabes, multiple sclerosis, progressive muscular atrophies, dystrophies, syringomyelia, epilepsy and hyperthyroidism. Disorders of speech were searched for as possibly indicating the presence of paresis.

A corroborated history of a mental disease requiring hospital treatment or observation was a cause for rejection. All cases showing symptoms of psychoses, of course, were rejected flatly. Men who were mental defectives or who suffered from psychoneuroses, or who were of a psychopathic character, and chronic inebriates and drug addicts were rejected.

In searching for mental defectives, the experience was novel. Many cases were referred for mental deficiency who were really not actually morons or imbeciles. They were illiterate, inexperienced, undeveloped, and had little or no schooling, due to the fact that they lived too long a distance from schools to be able to attend regularly, and also to the necessity of going to work at an early age on account of the economic conditions of the family, and a good many of them were below the average intelligence. They were examples of mental deficiency by deprivation. This class of men exhibited, after coming to camp, a slowing up of their mental processes, for obvious reasons. They received a multitude of new stimuli at once upon reaching camp, which was a new and unusual environment, in which many varied experiences crowded in on them at high speed, and they did not assimilate these readily at the start off. The result was that the individuals presented the appearances at first of mental retardation. Investigation of their social and industrial histories revealed, however, the fact that they had been able to get along moderately successfully in their home atmosphere, had saved money, owned their own places, were married and conducted their business successfully and we concluded that such cases were probably not mental defectives but that after a time they could develop into fairly good soldiers. These men were returned to their companies and in most instances the results were satisfactory.

It is not to be expected as a result of these examinations that the eliminating of all cases of nervous and mental disease will have been accomplished, but it is hoped that the number of casualties due to disturbances of the nervous system will be materially lessened. It would be too much to expect, however, that this number will be neglible.

The number of nervous and mental diseases reported up to February, 1918, is 13,481 from National Army and National Guard cantonments and the recruit depots. Of this number the diagnosis of mental deficiency was made in 35 o/o. There were over 12 o/o who were epileptics and over 11 o/o who were psychoneurotics. The diagnosis of constitutional psychopathic states was made in 9 o/o, and in 6 o/o the diagnosis of dementia praecox was made. Manic depressive insanity occurred in about 2 o/o of the cases and paresis in about 1 o/o of the cases. The remainder were made up of alcoholics, drug addicts, men suffering from organic nervous diseases and other psychoses than those mentioned. If we acknowledge that psychoneurotics and those showing constitutional psychopathic states are the most fruitful source of the cases of the war neuroses, we may expect that the elimination of these will have a definite result in lowering the number of such cases. It would be difficult to give an exact percentage of the whole number of drafted men examined who were rejected for nervous and mental disease, but roughly this may be estimated at 1.5 o/o, admitted to camps.

There were among the cases examined a group presenting symptoms referable to the nervous system which could be classified as exaggerations or malingers. These presented symptoms which have been observed and reported as occurring in the allied armies and in our own army in considerable numbers, as the result of trench warfare. The elimination of many of these cases no doubt also will lower the number of the cases of this type which we may expect to see and which were so frequent in the English and at first, at least, in the French armies.

In his experience in Camp Travis where about 40,000 men were examined or surveyed and where, as a member of the special board of medical examiners, the speaker saw all the cases of malingering; the most common type consisted of those whose complaints were exaggerations, or perseveration of symptoms which originated in a more or less severe injury some months or years previously, which gave rise to habitual attitude or limps similar to those described as occurring in the cases of war neuroses and now described by Babinski as reflex disorders or physiopathic disorders.

The limps were of very common occurrence. Some walked on the outside of the foot, due to a contraction of the anterior tibial muscle; or on the inside of the foot due to contraction of the extensors; or on the toes, a flexor contracture; or some walked with bent knee with a list to the same side.

Fake sciatic cases were frequent, as were also contractures of the trapezius muscle, the sterno-cleido-mastoid or all of the posterior group of neck muscles. The bent back, the camptocornia or plicature of the French was occasionally observed, also contractures of the arm, hand, and wrist. In fact, an observation which he was able to make as a result of his studies in Camp Travis, in the French hospitals, and in the British Shell Shock hospitals, as well as among our own soldiers subjected to shell fire, is that many of the nervous manifestations are presented by the drafted men in training camps, though in a minor degree, as is observed in the soldier on the firing line who has been under shell fire and who presents disorders referable to the nervous system. In other words, the war neuroses—or if you please shell shock phenomena—observed among the French and English soldiers and seen in the American Forces correspond to those nervous phenomena seen in training camps among drafted men upon their admission to camp or after they have been under training a short time, except, perhaps, in the latter case not to such an extreme degree.

